# Cross-sectional surveillance of Middle East respiratory syndrome coronavirus (MERS-CoV) in dromedary camels and other mammals in Egypt, August 2015 to January 2016

**DOI:** 10.2807/1560-7917.ES.2017.22.11.30487

**Published:** 2017-03-16

**Authors:** Mohamed Ali, Rabeh El-Shesheny, Ahmed Kandeil, Mahmoud Shehata, Basma Elsokary, Mokhtar Gomaa, Naglaa Hassan, Ahmed El Sayed, Ahmed El-Taweel, Heba Sobhy, Fasina Folorunso Oludayo, Gwenaelle Dauphin, Ihab El Masry, Abebe Wossene Wolde, Peter Daszak, Maureen Miller, Sophie VonDobschuetz, Emma Gardner, Subhash Morzaria, Juan Lubroth, Yilma Jobre Makonnen

**Affiliations:** 1 National Research Center, Division of Environmental Research, Giza, Egypt; 2 General Organizations of Veterinary Services, Ministry of Agriculture and Land reclamation (MoALR), Giza, Egypt; 3 Food and Agriculture Organization of the United Nations, Emergency Center for Transboundary Animal Diseases (ECTAD), Egypt; 4 EcoHealth Alliance, New York, New York, United States; 5 Food and Agriculture Organization of the United Nations, Rome, Italy; 6 Department of Veterinary Tropical Diseases, Faculty of Veterinary Science, University of Pretoria, South Africa

**Keywords:** MERS-CoV, Camel, Ruminants, Equines, bats, Egypt

## Abstract

A cross-sectional study was conducted in Egypt to determine the prevalence of Middle East respiratory syndrome coronavirus (MERS-CoV) in imported and resident camels and bats, as well as to assess possible transmission of the virus to domestic ruminants and equines. A total of 1,031 sera, 1,078 nasal swabs, 13 rectal swabs, and 38 milk samples were collected from 1,078 camels in different types of sites. In addition, 145 domestic animals and 109 bats were sampled. Overall, of 1,031 serologically-tested camels, 871 (84.5%) had MERS-CoV neutralising antibodies. Seroprevalence was significantly higher in imported (614/692; 88.7%) than resident camels (257/339; 5.8%) (p < 0.05). Camels from Sudan (543/594; 91.4%) had a higher seroprevalence than those from East Africa (71/98; 72.4%) (p < 0.05). Sampling site and age were also associated with MERS-CoV seroprevalence (p < 0.05). All tested samples from domestic animals and bats were negative for MERS-CoV antibodies except one sheep sample which showed a 1:640 titre. Of 1,078 camels, 41 (3.8%) were positive for MERS-CoV genetic material. Sequences obtained were not found to cluster with clade A or B MERS-CoV sequences and were genetically diverse. The presence of neutralising antibodies in one sheep apparently in contact with seropositive camels calls for further studies on domestic animals in contact with camels.

## Introduction

Since the first human case of Middle East respiratory syndrome coronavirus (MERS-CoV) in Saudi Arabia, in 2012, the World Health Organization (WHO) was notified of 1,698 laboratory-confirmed human cases and at least 609 human deaths from 26 countries as of March 2016 [1]. Primary infections have originated from countries within the Arabian Peninsula, but travel-associated cases and some secondary and nosocomial transmissions have been reported in other countries. A recent study in 2016 found antibodies against MERS-CoV in human serum in Kenya [2]. Available data from serological and molecular studies suggest that the primary source of MERS-CoV infection for many in the Arabian Peninsula appears to be dromedary camels [3-5]. Bats are also incriminated in the origins of many known mammalian coronaviruses including severe acute respiratory syndrome (SARS) [6,7]. The close relationship of MERS-CoV genome sequences and sequences of bat coronaviruses suggests that bats may be a reservoir for MERS-CoV [8]. Moreover, bat cell lines display the MERS-CoV specific receptor, dipeptidyl peptidase 4 (DPP4), and can be infected under experimental conditions [9]. Previous epidemiological studies to investigate the presence of MERS-CoV in bats found a close relationship between characterised sequences generated from bat faecal samples, and previously characterised MERS-CoV sequences [10-12]. 

A retrospective serological study conducted on 189 archived dromedary camels sera originating from main camel-exporting countries, Sudan and Somalia, in the period from 1983 to 1997, showed the presence of MERS-CoV neutralising antibodies in 81% of total samples suggesting long-term MERS-CoV circulation among camels [13]. Dromedaries from African countries (Egypt, Ethiopia, Kenya, Nigeria, Sudan, and Tunisia) and the Arabian Peninsula (Jordan, Oman, Qatar, Saudi Arabia, and United Arab Emirates) have high rates of MERS-CoV antibody seropositivity [14-20]. Dromedary camels are part of the culture of millions of people in Middle Eastern countries where camel milk and meat are consumed. Most dromedary camels traded in the Middle East are bred in East African countries, primarily in Ethiopia, Kenya, Somalia, and Sudan [21]. During the last 5 to 6 years (2010 to 2015), over 1.2 million camels were imported to Egypt, nearly 70% from Sudan and the rest from the African Horn, mainly Ethiopia [22].

Serological investigations carried out on camels in Egypt, revealed high levels of antibodies against MERS-CoV [17,23]. Furthermore, MERS-CoV was detected virologically in specimens collected from abattoirs in the country [23]. The objectives of this study were to determine the prevalence of MERS-CoV in imported and resident camels and investigate the prevalence of the virus among other domestic animals in Egypt.

## Methods

### Study animals and sampling strategy

**Figure 1:**
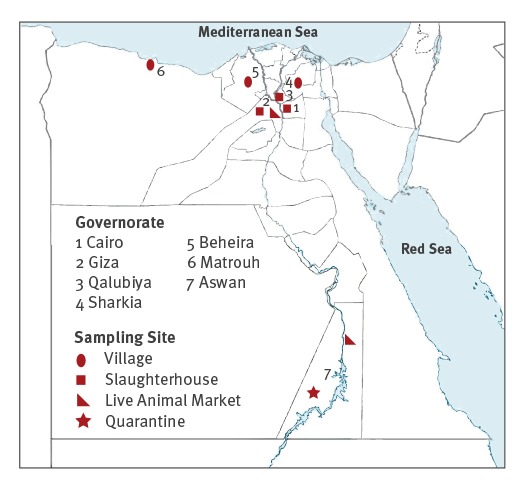
Site map of the collected samples from dromedary camels and domestic animals in Egypt, August 2015–January 2016 (n =1,223 animals^a^)

^a^ In addition to 1,078 camels. a total of 145 domestic animals were sampled and included cattle (n = 35), sheep (n = 51), goats (n = 36), donkeys (n = 15), buffaloes (n = 4) and horses (n = 4).

A total of 1,176 sera and 1,223 nasal swabs, were collected from 1,223 animals including 1,078 dromedary camels (339 resident and 739 imported) and 145 other domestic animals (cattle, n = 35; sheep, n = 51; goats, n = 36; donkeys, n = 15; and buffalo and horses, n = 4 each) from different sampling sites (quarantine posts, live animal markets, slaughterhouses and villages) from seven governorates of Egypt (Figure 1) between August 2015 and January 2016.

Milk samples (3–5mL; n=38) and rectal swabs (in 1mL viral transport media; n=13) were also sampled from resident camels in a village located in the Matrouh governorate. 

In addition, 109 throat swabs and 91 sera were collected from 24 fruit bats (*Rousettous aegyptiacus)* and 85 insectivorous bats (*Pipistrellus deserti*, n = 28; *Nycteris thebaica*, n = 30; *Taphozous perforates*, n = 27) from Abo Rawash, Giza governorate, and included in the study. 

A multistage sampling strategy involving a combination of simple stratified (for sex and age) and systematic sampling was employed to obtain samples from camels. Origin of camels was identified at the place of quarantine in Egypt, or from information obtained from the owners. Camels less than two years of age were considered young while those over two years-old were considered adult. Since the majority of the imported camels were adult male, purposive sampling was employed to include female adult camels particularly in the resident camels. Sampling procedures were approved by the Ethics Committee of the National Research Centre, Egypt.

The nasal, throat, rectal swabs and milk were analysed using molecular virological techniques.

### Serological testing

Serum microneutralisation assay was conducted as described [17], using Vero-E6 cell monolayers. Briefly, twofold serial dilutions of 200μL heat-inactivated sera (56 °C for 30 min) were made, starting with a dilution of 1:10. The serum dilutions were mixed with equal volumes of 200 tissue culture infectious dose (TCID_50_) of dromedary MERS-CoV Egypt NRCE-HKU270 (Egypt 270). After 1 hour of incubation at 37 °C, 35 μL of the virus–serum mixture were added in quadruplicate to Vero-E6 cell monolayers in 96-well microtitre plates. After 1 hour of adsorption, an additional 150 μL of culture medium were added to each well. The plates were then incubated for three more days at 37 °C in 5% CO_2_ in a humidified incubator. Virus back-titration was performed without immune serum to assess input virus dose. Cytopathic effect (CPE) was read at 3 days post infection. The highest serum dilution that completely protected the cells from CPE in half of the wells was taken as the neutralising antibody titre and was estimated using the Reed–Muench method. Positive cut off points was set at values greater or equal to 1:20 serum dilution points.

### Real-time reverse transcription-PCR

Real-time reverse transcription-PCR (rtRT-PCR) targeting upstream of the envelope protein gene (UpE) of MERS-CoV was used for screening [24]. Confirmation was made using the open reading frame (ORF) 1a, RNA-dependent RNA polymerase (RdRp) or nucleocapsid protein (N) gene, based on the recommendation of World Health Organization for MERS-CoV diagnosis [25]. Briefly, 5 µL of extracted RNA was subjected to rtRT-PCR using UpE primers described elsewhere [24]. The rtRT-PCR was performed using a Verso One Step rtRT-PCR Kit according to the manufacturer’s protocol. All positive samples by the UpE assay regardless of cycle threshold (Ct) value were then confirmed by one of ORF1a, RdRp, or N gene RT-PCR assay as described previously [24,26]. PCR products were analysed by sequencing using the protocol available on the web (on line Technical Appendix: http://wwwnc.cdc.gov/eid/article/20/6/14-0299-techapp1.pdf).

#### Reverse transcription-PCR for MERS-CoV genotyping

A partial 640 bp fragment of the spike gene was amplified using 50-Fwd (5’-CCAATTTA-CGCCAGGATGAT-3’) and 50-Rev (5’-AATAGAGGCGG AAATAGCAC-3’) primers in the first round using one step RT-PCR kit (QIAGEN) and a total reaction volume of 25 µL including 5 µL of 5X reaction buffer, 1 µL dNTPs, 1 µL enzyme mix, 1.5 µL (10 pmol) forward primer, 1.5 µL (10 pmol) reverse primer, 10 µL ddH_2_O and 5 µL of sample RNA. Subsequent to thirty min at 50 °C and 95 °C for 15 min, the RT-PCR also comprised 45 cycles of 94 °C for 15 s, 55 °C for 30 s and 72 °C for 60 s followed by a final step of 72 °C for 10 min. The PCR product was then submitted to a second PCR round using the same primers as in the first round and Phusion High Fidelity PCR Master Mix Kit (Thermo Scientific). The PCR had a 25 µL reaction volume, with 12.5 µL of 2 X phusion master mix, 1.5 µL (10 pmol) forward primer, 1.5 µL (10 pmol) reverse primer, 7.5 µL H_2_O and 2 µL of the first round PCR product. The PCR cycler conditions were 98 °C for 30 s then 45 cycles (98 °C for 10 s, 55 °C for 30 s, 72 °C for 60 s), then 72 °C for 10 min. The final PCR product was gel purified and subsequently sequenced with the same primers at the Macrogen sequencing facility (Macrogen, South Korea). One positive imported sample (NC2603/2015) from Sudan was subjected to whole genome sequencing according to a previously published procedure [27]. The phylogenetic tree was constructed using MEGA6 programme [28].

### Data management and analysis

Data collected from the study animals were coded and entered in a Microsoft excel sheet. All statistical analyses were performed using SPSS version 16 for windows. The association between MERS-CoV prevalence in camels and the study variables (sampling site, origin, age and sex) were analysed by Pearson chi-squared test of independence. Statistical significance was considered at p- value less than 0.05.

## Results

### Serological analysis

Of the 1,031 camels, which were serologically tested, 871 (84.5%) had MERS-CoV neutralising antibodies in their sera (Table 1). 

**Table 1:** MERS-CoV surveillance test results in camels based on origin, Egypt, August 2015–January 2016 (n = 1,078 camels^a^)

Camel origin	Microneutralisation test			CMLE OR^b^ (95% CI)	P value (for OR)	P value (for hypothesis)	rtRT-PCR			P value (for hypothesis)
	Number tested	Number of camels positive	Per cent positive				Number tested	Number of camels positive	Per cent positive	
East Africa	98	71	72.4%	0.84 (0.51–1.41)	0.50	p < 0.001 χ2 = 53.24	115	4	3.5%	p < 0.001 χ2 = 15.246
Sudan	594	543	91.4%	3.39 (2.24–4.98)	< 0.0001		623	35	5.6%	
Egypt (resident)	339	257	75.8%	1.00	Ref.		340	2	0.6%	
**Total**	**1,031**	**871 **	**84.5%**	NA	NA	**NA**	**1,078**	**41 **	**NA**	**NA**

CI: confidence interval; CMLE: conditional maximum likelihood estimate; MERS-CoV: Middle East respiratory syndrome coronavirus; NA: not applicable; OR: odds ratio; ref.: reference; rtRT-PCR: real-time reverse transcription PCR.

^a^ Of 1,078 camels, a subset of 1,031 underwent serum testing for MERS-CoV antibodies by microneutralisation assays, while all were sampled for rtRT-PCR testing.

^b^ CMLE OR is the conditional maximum likelihood estimate of the odds ratio based on Mid-P exact confidence interval.

The seroprevalence was significantly higher in imported (614/692; 88.7%) than in resident camels (75.8%; Table 1) (p < 0.05). Based on the area of origin, seroprevalence varied significantly among camels originating from East Africa, Sudan, and Egypt and was 72.4%, 91.4%, and 75.8%, respectively (p < 0.05). Camels sampled from live animal markets, quarantine facilities, slaughterhouses, and villages had seroprevalence of 94.5%, 95.7%, 77%, and 75% respectively and the differences was significant (p < 0.05 Table 2). Overall, adult camels had significantly higher seroprevalence (87.3%) than young camels (51.8%) (p<0.001). A significantly higher seropositivity was observed for camels from the live animal markets (OR = 5.52; p < 0.0001) and quarantine facilities (OR = 7.25; p < 0.0001) as compared with those from villages and the slaughterhouses.

**Table 2:** MERS-CoV surveillance test result in camels based on sampling site, age and sex, Egypt, August 2015–January 2016 (n = 1,078 camels^a^)

Category	Microneutralisation test			CMLE OR^b^ (95% CI)	P value (for odd ratio)	P value (for hypothesis)	rRT-PCR			P value (for hypothesis)
	Number tested	Number positive	Per cent positive				Number tested	Number positive	Per cent positive	
**Sampling site**										
Live animal market	289	273	94.5%	5.52 (3.20–9.96)	< 0.0001	p < 0.001 χ2 = 67.47	290	9	3.1%	p < 0.001 χ2 = 31.97
Village/Egypt	339	256	75.8%	1.00	Ref.		340	2	0.6%	
Quarantine	164	157	95.7%	7.25 (3.42–17.42)	< 0.0001		164	4	2.4%	
Slaughterhouse	239	184	77%	1.09 (0.73–1.61)	0.69		284	26	9.2%	
Total	1,031	871	84.5%	NA	NA	NA	1,078	41	3.8%	NA
**Age**										
Young	81	42	51.8%	1.00	Ref.	p < 0.001 χ2 = 71.39	82	2	2.4%	p = 0.77 χ2 = 0.53
Adult	950	829	87.3%	6.34 (3.93–10.24)	< 0.0001		996	39	3.9%	
**Sex**										
Male	765	651	85.1%	1.19 (0.82–1.73)	0.35	p = 0.38 χ2 = 0.86	798	21	2.6%	p < 0.001 χ2 = 13.07
Female	266	220	82.7%	1.00	Ref.		280	20	7.1%	

CI: confidence interval; CMLE: conditional maximum likelihood estimate; MERS-CoV: Middle East respiratory syndrome coronavirus; NA: not applicable; OR: odds ratio; ref.: reference; rtRT-PCR: real-time reverse transcription PCR.

^a^ Of 1,078 camels, a subset of 1,031 underwent serum sampling for MERS-CoV antibodies by microneutralisation assays, while all were sampled for rtRT-PCR testing.

^b^ CMLE OR: Conditional maximum likelihood estimate OR based on Mid-P exact confidence interval.

Both male and female camels had a comparable (p > 0.05) level of seroprevalence (85.1% and 82.7% respectively), and risk of seropositivity (Table 2). Tested samples from 126 ruminants (cattle, sheep, goats, and buffaloes) and 19 equines (donkeys and horses) were negative for neutralising MERS-CoV antibodies but one serum sample from a sheep had 1:640 neutralising titre. None of the 91 tested bats was positive for MERS-CoV neutralising antibodies.

### Virus genomic detection

Of the 1,078 nasal samples from camels, 41 (3.8%) were positive for MERS-CoV using MERS-CoV PCR tests indicating the presence of active or passive viral infection. Of the 41 positive camels, four originated from East Africa, 35 from Sudan and the other two from the study sites in Egypt (Table 1). The confirmed PCR-positive MERS-CoV cases was significantly higher in females than males (p < 0.001). All the 38 milk samples and 13 rectal swabs were negative for MERS-CoV. Similarly, the 145 nasal swabs from domestic ruminants and equines were negative for MERS-CoV. Throat swabs collected from 109 bats were negative for MERS-CoV.

### Sequence analysis

**Figure 2:**
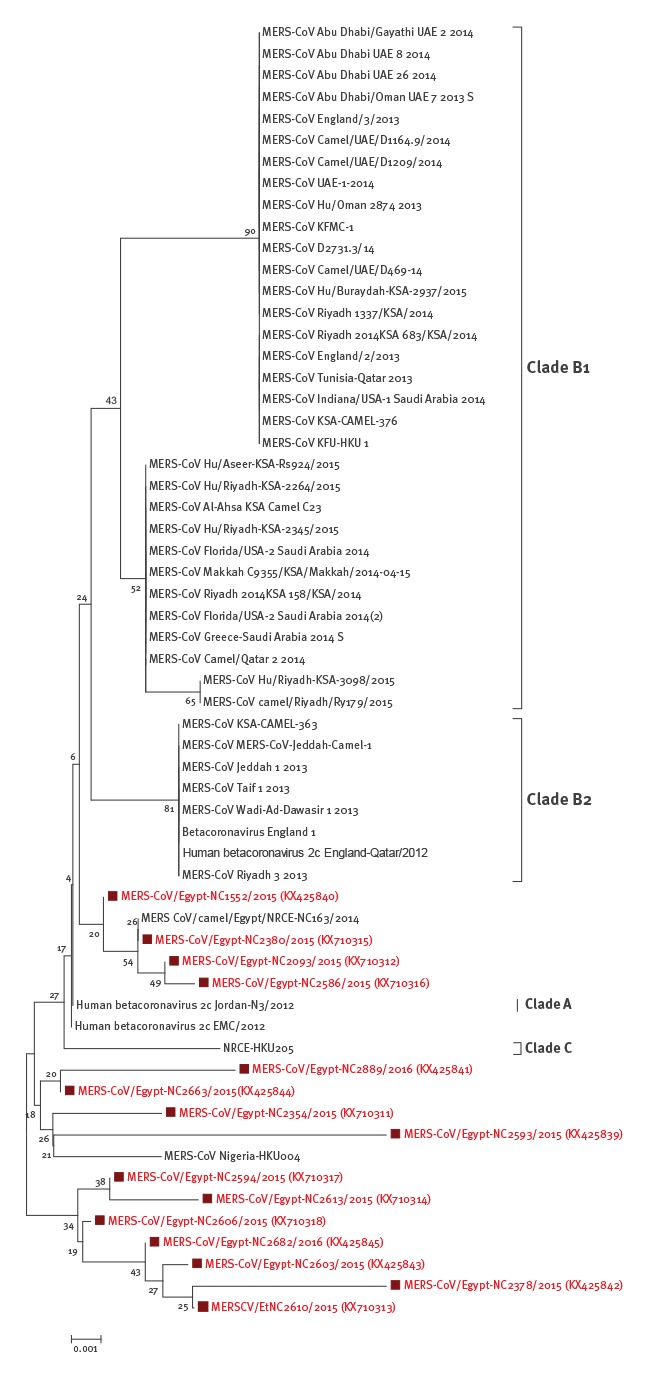
Phylogenic analysis of partial MERS-CoV spike sequences retrieved from dromedary camels residing in or imported to Egypt from Sudan between August 2015 and January 2016

**Figure 3:**
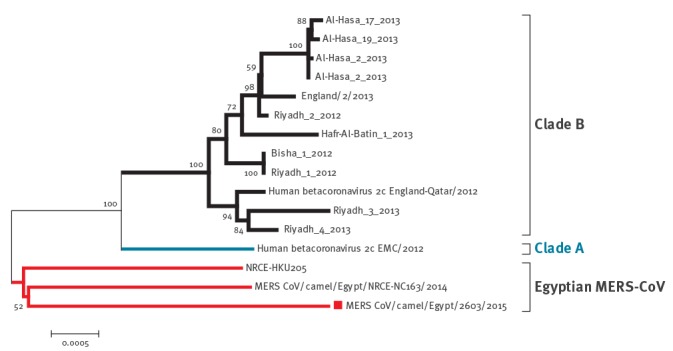
Phylogenic analysis of a full MERS-CoV genome sequence retrieved from an imported dromedary camel from Sudan between August 2015 and January 2016

A phylogenetic tree was compiled based on partial spike nucleotide sequences obtained from 15 strongly positive samples. The sequences were derived from one camel residing in Egypt as well as from camels imported from Sudan, which had been sampled in a slaughterhouse (n = 9) and live animal markets (n = 5). The tree suggested that sequences from camels investigated in Egypt formed separate groups from previously published sequences of MERS-CoV (Figure 2). Moreover, a phylogenetic analysis of full genomes showed that sequences from camels sampled in Egypt were genetically diverse and clustered neither with clades A or B (Figure 3).

Representative viruses from clades A, B and C are indicated and marked with vertical bar. Phylogenetic analysis was done using the neighbour-joining algorithm with the Kimura two-parameter model. The tree is drawn to scale, with branch lengths in the same units as those of the evolutionary distances used to infer the phylogenetic tree. The reliability of phylogenetic inference at each branch node was estimated by the bootstrap method with 1,000 replications; evolutionary analysis was conducted in MEGA 6.06. Viruses sequenced for this study are marked with red squares.

Representative viruses from the two major MERS-CoVs clades (A and B) are indicated and marked with vertical bar. Phylogenetic analysis was done using the neighbour-joining algorithm with the Kimura two-parameter model. The reliability of phylogenetic inference at each branch node was estimated by the bootstrap method with 1,000 replications. The tree is drawn to scale, with branch lengths in the same units as those of the evolutionary distances used to infer the phylogenetic tree. Evolutionary analysis was conducted in MEGA 6.06. The virus sequenced for this study is marked by a red square.

## Discussion

The present study demonstrated that most of the camels that were imported to Egypt were seropositive for MERS-CoV (88.7%; 614/692) and virus genetic materials was detected in 5.3% (39/738) of the imported camels. The origins of the camels were Sudan and East Africa. Surprisingly, no human cases of MERS CoV infection has been recorded among camel traders from these countries. This may be due to the lack of diagnostic tools and experience for virus detection or maybe due to the rarity of virus transmission from camels to humans.

Data from experimental camel infections suggest that MERS-CoV is a mild respiratory infection in camels [29] and although camels previously sampled at abattoirs shed the virus, they did not have overt clinical symptoms [23]. Egypt imports large numbers of live camels each year to meet its animal protein demand. According to the Ministry of Agriculture, almost 70% of the imported camels during the past five years originated from the Sudan and the rest from East Africa, mainly Ethiopia. These imported camels are quarantined usually for 2–3 days at the point of entry before they gain entry for sale at live animal markets. The animals often travel long distances by trucks and may be moved from one live animal market to another. Transport stress and close vicinity of camels during transport may precipitate disease dissemination, particularly in animals with latent infection and carrier animals, while transmission may be facilitated spatio-temporally in the different markets. The high MERS-CoV seroprevalence both in resident and imported camels and the presence of active viral infection circulating in the country were indications that the virus may have become ubiquitous in Egypt. Inter-market movement and transport stress may partially explain the higher seropositivity and molecular analysis results in samples obtained from the live animal markets, quarantine facilities, and the slaughterhouses.

Testing of archived dromedary sera has revealed that MERS-CoV has been circulating for at least three decades and is not a newly emerged virus, but rather a virus that has only recently been discovered [3,13,15]. Results of study in Egypt published in 2014 showed that 93.6% of camels originating from Sudan were seropositive for MERS-CoV, a finding is consistent with the present study where 91.4% of camels imported from that country were seropositive [23].

Analysis of the results based on age showed that adult camels had higher seroprevalence of MERS-CoV antibodies (87.3%) compared with young camels (51.8%) (p < 0.05). The variation might be due to the small number of young camels tested or the higher likelihood of exposure of adult camels. In addition, young camels have been more acutely infected in past studies and may have died rather than seroconverted [18]. Similar studies elsewhere also indicated a higher seroprevalence in adult than in juvenile camels [30]. Although the number of seropositive samples was comparable in female and male camels, the number of confirmed PCR positive MERS-CoV animals was significantly higher in females than males (p < 0.05). There was however no significant difference in rtRT-PCR positive cases between the age groups.

Nucleotide sequencing of the amplicons from 15 of 41 PCR-positive samples for MERS-CoV genetic material, followed by phylogenetic analysis showed that the sequences recovered in the current study in Egypt were distinct from those in clade A and B. This was also the case for previously identified MERS-CoV sequences derived from camels in Egypt (e.g. MERS CoV/camel/Egypt/NRCE-NC163/2014) [31] which were distinct from MERS-CoV EMC/2012 isolate [23].

All the 145 domestic animals (ruminants and equines) tested for MERS-CoV genetic materials were negative, in agreement with previous studies conducted in Jordan and Egypt [19]. Except one sheep, all domestic animals serologically tested were negative. Similarly, previous serological studies conducted on goats, sheep, and cows were all negative [19]. Also according to a prior report, 25 cows and eight buffalo from Egypt tested negative to MERS-CoV neutralising antibodies [17]. The seropositive sheep found in the current study was apparently in contact with seropositive camel herds in villages. This finding is significant and adds to the knowledge of host range of MERS-CoV. The DPP4 receptor for MERS-CoV has been found to be present in camel, goat, cow and sheep [32], and Reusken et al. [19] have earlier confirmed that six sheep reacted to MERS-CoV antigens but without neutralising antibodies [19]. Further and extensive studies on domestic animals especially in those in contact with camels are required to elucidate the possibility of MERS-CoV transmission from camels to such animals.

Whereas MERS CoV has been found in one bat sample in Saudi Arabia [5], all the 109 bats in the present study, were negative for MERS-CoV using both serology and molecular assays. Bats have been incriminated as the origin of many known mammalian coronaviruses including SARS [7]. A 190 nt RNA fragment of MERS-CoV was detected in a bat faecal sample [11]. However, since human–bat contact is limited, camels have been more implicated as a probable intermediate host [33].

In conclusion, the very high prevalence of MERS-CoV neutralising antibodies in both resident and imported camels indicates the widespread and ubiquitous presence of the virus in the country. A systematic longitudinal study, however, is needed to follow up imported camels from their country of origin until they reach the slaughterhouses to understand the epidemiology of the disease along the camel market chain. A separate study on resident camels is needed to understand the dynamics of infection in local camels as opposed to in imported camels. The very high seroprevalence detected in camels warrants the initiation of an active surveillance study on humans, particularly those that are at higher risks of exposure to MERS-CoV infections such as camel traders and abattoir workers.
