# Authors’ correction for Euro Surveill. 2017;22(11)

**DOI:** 10.2807/1560-7917.ES.2017.22.19.30531

**Published:** 2017-05-11

**Authors:** Eurosurveillance editorial team

**Affiliations:** 1 European Centre for Disease Prevention and Control (ECDC), Stockholm, Sweden

In the article ‘Cross-sectional surveillance of Middle East respiratory syndrome coronavirus (MERS-CoV) in dromedary camels and other mammals in Egypt, August 2015 to January 2016‘ by Ali et al., published on 16 March 2017, the order of Dr Folorunso Oludayo Fasina’s names was wrong in the authors’ list, leading to abbreviation as FF Oludayo instead of FO Fasina. This was corrected on 17 March 2017. Moreover at the request of all authors, the name of Emma Gardner was removed from the author list. This change was done on 11 May 2017.

